# The Atomic Partial Charges Arboretum: Trying to See the Forest for the Trees

**DOI:** 10.1002/cphc.202000040

**Published:** 2020-03-23

**Authors:** Minsik Cho, Nitai Sylvetsky, Sarah Eshafi, Golokesh Santra, Irena Efremenko, Jan M. L. Martin

**Affiliations:** ^1^ Department of Organic Chemistry Weizmann Institute of Science 76100 Rehovot Israel; ^2^ Present Address: Department of Chemistry Brown University Providence Rhode Island 02912 USA; ^3^ Present Address: Integrated Science Program McMaster University Hamilton Ontario L8S 4 M1 Canada

**Keywords:** atoms in molecules, charge distributions, molecular modelling, population analysis, principal components of ionicity

## Abstract

Atomic partial charges are among the most commonly used interpretive tools in quantum chemistry. Dozens of different ‘population analyses’ are in use, which are best seen as proxies (indirect gauges) rather than measurements of a ‘general ionicity’. For the GMTKN55 benchmark of nearly 2,500 main‐group molecules, which span a broad swathe of chemical space, some two dozen different charge distributions were evaluated at the PBE0 level near the 1‐particle basis set limit. The correlation matrix between the different charge distributions exhibits a block structure; blocking is, broadly speaking, by charge distribution class. A principal component analysis on the entire dataset suggests that nearly all variation can be accounted for by just two ‘principal components of ionicity’: one has all the distributions going in sync, while the second corresponds mainly to Bader QTAIM vs. all others. A weaker third component corresponds to electrostatic charge models in opposition to the orbital‐based ones. The single charge distributions that have the greatest statistical similarity to the first principal component are iterated Hirshfeld (Hirshfeld‐I) and a minimal‐basis projected modification of Bickelhaupt charges. If three individual variables, rather than three principal components, are to be identified that contain most of the information in the whole dataset, one representative for each of the three classes of Corminboeuf et al. is needed: one based on partitioning of the density (such as QTAIM), a second based on orbital partitioning (such as NPA), and a third based on the molecular electrostatic potential (such as HLY or CHELPG).

## Graphical abstract

**Forest of ionicity**: The dozens of different population analysis (POP) techniques in existence leave the end‐user perplexed. Through principal component analysis on a sample of over 2,000 main‐group molecules, it emerges that just three features account for nearly all the variation: a “general ionicity” that has all POPs going in sync, a second component that pits QTAIM (plus APT) against the rest, and a weaker third that has ESP‐ vs. orbital‐based POPs.

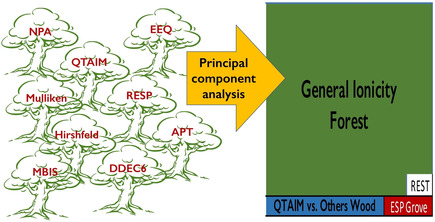

## 1 Introduction

Atomic partial charges *q*_A_ are a central concept in general chemistry. Unfortunately, they do not correspond to a single, well‐defined, quantum mechanical observable. The very notion of atomic partial charges, however, does imply that “atoms in molecules” (in the broad sense of the word, not the narrow one of Bader/QTAIM^1^ charges) are meaningful concepts.

Parr, Ayers, and Nalewajski^2^ consider partial charges to be *noumena*: The term noumenon, originally coined by Plato from the Greek word *noös* (knowledge, cognition), is defined by the Oxford English Dictionary as “an object knowable by the mind or intellect, not by the senses; specifically (in Kantian philosophy) an object of purely intellectual intuition”. Frenking and Krapp^3^ speak of “unicorns”: “my[th]ical animal[s] whose appearance is known to everybody although nobody has ever seen one”.

In reaction, Matta and Bader^4^, ^5^ strongly took exception. They point out that Bader's QTAIM charges, in particular, can be seen as expectation values of well‐defined operators, namely: Heaviside “step” functions that are unity within one set of zero‐flux surfaces and zero outside. The fact that QTAIM charges often are at odds with “chemical intuition” (or at least chemical received wisdom) leaves the reader even more perplexed, and more in need of a guide.^6^

Perhaps a third term is more apropos here, namely the statistical one “proxy variable”. A proxy variable is a (fairly easily) measurable quantity that acts as an indirect “proxy” for a deeper concept that eludes direct measurement. Examples from economics are proxies for the standard of living such as GDP per capita or PPP (purchasing power parity). Other examples, from psychology, are IQ scores when used as proxies for general intelligence, or (relatedly) SAT scores as predictors for scholastic success. (As a hoary quip goes, the only thing standardized cognitive tests directly measure is performance on those tests.) In that sense, partial charges are “proxy variables” for a deeper chemical concept that Meister and Schwarz^7^ have termed “[molecular] ionicity”.

Corminboeuf and coworkers^8^ as well as Cramer and Truhlar^9^ have attempted taxonomies of breeds within this particular species of “chemical unicorn”. Corminboeuf et al. distinguish three broad classes of computed partial charges:

1. fitting to an observable quantity such as the molecular electrostatic potential on the van der Waals surface, such as done in CHELP^10^ (charges from electrostatic potentials), CHELPG^11^ (ditto using a grid), Merz‐Kollman,^12^ and HLY^13^ (Hu−Lu‐Yang) charges;
2. partitioning in terms of atomic orbitals, such as done in Mulliken charges^14^ and their variants, Natural Population Analysis (NPA),^15^ and Intrinsic Bond Orbitals,^16^ (IBO, also known^17^ as Quasi‐Atomic Natural Bond Orbitals^18^);
3. atoms‐in‐molecules partitioning of the electron density, either into disjoint QTAIM^1^ or Voronoi^19^ cells, or into overlapping “stockholder domains” (such as the original Hirshfeld population analysis^20^, ^21^ and its manifold offspring, e. g.,^20^, ^21^, ^22^, ^23^, ^24^, ^25^, ^26^)^1^.

In a separate category they place “experimental” charges, which are measured (or rather, for which trends are inferred) from NMR or EXAFS chemical shifts, dipole moments, and the like.

Cramer and Truhlar^9^ proposed a slightly different taxonomy which, with variations, we will adhere to in the present work.

- Class I charges are derived from experimentally measurable properties, e. g., from observed deformation densities (as in the work of the late Philip Coppens^27^, ^28^), from the electronegativity equalization principle, or from, e. g., dipole moments of diatomic and small (usually highly symmetric) polyatomic molecules.
- Class II charges are extracted from the molecular orbitals (Mulliken, NPA,…) or the electron density (Bader QTAIM, Hirshfeld,…).
- Class III charges are extracted from the wave function or electron density by fitting a physical observable (e. g., the electrostatic potential) derived from it.
- Class IV charges, such as the CM5 model introduced by Truhlar and coworkers,^29^ are based on semiempirical adjustment of a well‐defined Class II or Class III model to better reproduce one or more physical observables (e. g. the dipole moment).

(We note in passing that the legal fiction known as “formal charges” might be termed Class 0.)

A number of review articles have been devoted to partial charges, such as Wiberg and Rablen,^30^, ^31^ Bachrach,^32^ Cramer and Truhlar,^9^ and most recently Ayers and coworkers^26^ (which focuses principally on Hirshfeld‐type approaches from an information theoretical point of view).

Normally, “observability” in the quantum mechanical sense of the word implies the existence of an operator for which one can obtain an expectation value. Cioslowski and Surjan^33^ propose a weaker, more general definition where a uniquely defined computational protocol that extracts uniquely defined numerical values from the wave function, which have well‐defined infinite basis set limits, is said to satisfy a “generalized observability criterion”. (It should be pointed out that the hoary Mulliken population analysis,^14^ for instance, does not satisfy said criterion on account of its pathological basis set dependence – nor do modified Mulliken analyses like Löwdin,^34^ Ros‐Schuit,^35^ or Bickelhaupt.^36^ However, minimal basis set projections^37^ – denoted here as MBS‐Mulliken,^14^, ^37^ MBS‐Bickelhaupt (Ref. [36] and vide infra), etc. – do satisfy the Cioslowski‐Surjan criterion.^36^)

So, there are now several dozen charge distributions “on the market”. But how distinct are they really? How different a picture do they really paint? Answers to this question might be found in feature reduction techniques – most notably from the best‐known such technique, PCA (principal component analysis).

Investigating this question requires a dataset of molecules large and chemically diverse enough that any conclusions reached cannot (easily) be dismissed as sample selection artefacts. The almost 2,500 unique species in the GMTKN55 benchmark^38^ (general main‐group thermochemistry, kinetics, and noncovalent interactions, 55 problem subsets) for density functional methods are one useful starting point. (See Refs.^38^, ^39^, ^40^, ^41^, ^42^ for example applications to DFT.)

We shall show that there is indeed a “method in the madness” and that almost all of the data variation can be represented by two to three principal components.

## Computational Methods

### Population Analyses Considered

We considered the following population analyses as representatives of the different classes in the Cramer and Truhlar^9^ taxonomy (where we shall introduce some subclasses below):

(1) for Class I charges, the EEQ (electronegativity equalization principle) charges as implemented in the DFTD4 program of the Grimme group (Section II.A of Ref. [43]);

(2) Class II is subdivided here into:

 •Class IIa: from partitioning orbitals. (NB: this effectively corresponds to Corminboeuf's Class 2.) We can subdivide further into:

  – IIa1: Mulliken^14^ and variants (Löwdin,^34^ Bickelhaupt,^36^…) applied in the full basis set. We considered these but discarded them on account of their pathological basis set dependence.

  – IIa1 M: projection of the MOs onto a minimal basis set^37^ followed by the above techniques. This eliminates the basis set hypersensitivity and leads to values that satisfy the extended Cioslowski‐Surjan observability criterion.^33^ We here consider MBS‐Mulliken^37^ and (by straightforward extension) MBS‐Bickelhaupt. The latter differs from MBS‐Mulliken in that off‐diagonal overlap populations are taken into account by diagonal‐overlap weighted average instead of simple average:

    $Q_{ii,Bickelhaupt}^{{}^{'}}=q_{ii}+\sum_{i\neq j}\left(\frac{q_{ii}}{q_{ii}+q_{jj}}\right)\left(q_{ij}+q_{ji}\right)$

    $Q_{ii,Mulliken}^{{}^{'}}=q_{ii}+\frac{1}{2}\sum_{i\neq j}\left(q_{ij}+q_{ji}\right)$

  – IIa2: techniques based on some form of natural or intrinsic atomic orbital: here we consider Weinhold's NPA (natural population analysis^15^) and the IAO (intrinsic atomic orbitals) of Knizia,^16^ which are functionally equivalent^17^ to Ruedenberg's QUAOs (quasi‐atomic orbitals^18^, ^44^).

 •Class IIb: from partitioning the electron density into atomic domains & integrating over those. (This effectively corresponds to Class 3 in Corminboeuf's taxonomy.) We can further distinguish between fuzzy and discrete domains.

  – IIb1: fuzzy domains: we primarily focus on variants of the “stockholder” population analysis of Hirshfeld,^20^, ^21^ specifically:

 •his original method (in which the deformation density is partitioned based on the promolecular density);

 •the iterated Hirshfeld^22^ (Hirshfeld‐I) method, where the proatom densities that define the promolecular density are iteratively interpolated between ionization states;

 •the Iterative Stockholder Approach^23^ (ISA) in which proatoms are avoided through angular integration around an atom;

 •MBIS (minimal basis iterative stockholder^25^), which mitigates certain issues with proatoms that are unbound in vacuo (e. g., N^–^, N^2–^, N^3–^);

 •Finally, the DDEC6 (density derived electrostatic and chemical approach, version 6) of Manz^[24](a‐f)^ which is designed for computational resilience in problematic systems and contains many adjustments to ensure proper behavior in solids^2^. This approach has been further developed for additional properties (e.g., bond orders, ^[24](c)^ dispersion coefficients^[24](e)^) that are beyond the scope of the present paper.

Stockholder‐type charges have been rationalized based on information theory^2^, ^45^ and were the subject of a recent review.^26^ (We considered Salvador's topological fuzzy Voronoi charges^46^ for a representative sample consisting of the W4‐17 benchmark,^47^ and found said charges to be very similar in practice to Bader QTAIM charges, R^2^=0.97.)

  – IIb2: disjoint/discrete domains: the most important representative of this are QTAIM (quantum theory of atoms in molecules^1^), a.k.a. “Bader charges”, in which zero‐flux surfaces are used to partition the electron density into atomic cells within which numerical integration takes place.

Maslen and Spackman^48^, ^49^ proposed an modification inspired by Hirshfeld charges: while zero‐flux surfaces are determined as in QTAIM, the integration is performed instead on the deformation density (i. e. ρ_molecule_ – ρ_promolecule_). This mitigates the tendency of QTAIM to amplify charges: zero‐flux surfaces tend to run far from high electronegative atoms like O and F, at the expense of less electronegative atoms.

The Voronoi Deformation Densities (VDD) of Fonseca‐Guerra et al.,^19^ go one step further, in that they partition the deformation density through simple Voronoi tessellation: any point in space closer to a given atom than to the others is assigned to that atom.

(3) Class III can be further subdivided into:

 •Class IIIa: based on the electrostatic potential. (This corresponds to Class 1 in Corminboeuf's taxonomy.) The original CHELP of Chirlian and Francl^10^ was modified by Breneman and Wiberg (CHELPG)^11^ to improve rotational invariance. Merz‐Kollman (a.k.a. Merz‐Singh‐Kollman^12^, ^50^), Hu−Lu‐Yang (HLY^13^), and RESP (restrained electrostatic potential^51^) all have the same physical basis but merely represent different integration grid specifics for the ESP. As HLY appears to be the most stable numerically and is available for all elements represented in our dataset, we have focused primarily on HLY.

 •Class IIIb: based on other electrostatic properties. Here we consider Cioslowski's APT (atomic polar tensor^52^) charges, based on the trace of the dipole moment derivatives.

(4) under Class IV we consider CM5 (charge model 5),^29^ an empirical adjustment of Hirshfeld to better reproduce molecular dipole moments, and ADCH:^53^ (atomic dipole corrected Hirshfeld), an adjustment to ensure reproduction of molecular dipole moment obtained at the same calculation level.

In addition, we consider ACP, atomic charge partitioning^54^ and especially its iterative variant i‐ACP,^55^ which arguably can be pigeonholed both under Class IV (on account of their adjustment for better electrical properties) and under IIb1 (as they bear an obvious kinship to MBIS in representing proatoms by Slater‐type functions).

## Other Computational Details

All electronic structure calculations were carried out using the PBE0 functional^56^, ^57^ with the def2‐TZVPP basis set^58^ using the Gaussian,^59^ MOLPRO,^60^ and Q‐CHEM^61^ electronic structure program systems. SG‐3^62^ or equivalent integration grids (e. g. Grid=UltraFine in Gaussian) were employed.

The reference geometries were obtained from the online supporting information to the GMTKN55 paper and used without further optimization. Species with trivial charge distributions (e. g. atoms, homonuclear diatomics, tetrahedral P_4_,…) were removed from the dataset, as were the handful of duplicate species, which left 2,125 unique molecules. Details of the electronic structure and postprocessing software used to generate each specific set of partial charges are given in Table 1. The various aspects of this process were automated using a collection of scripts developed in‐house. For easy data manipulation, Python scripts using numpy and pandas libraries were written. Duplicates were identified from the input geometries (by comparison of the rotational constants). Holes in the data were generally from technical limitations dealing with molecules that contain heavy elements for which some parameter was lacking in the available implementation.

**Table 1:** Details of software used for various population analyses.

Software	Atomic Partial Charges
Multiwfn^63^	Atomic Dipole Corrected Hirshfeld (ADCH),^53^ Voronoi Deformation Density(VDD),^19^ Becke,^64^ Ros‐Schuit,^35^ CHelpG (CHarges from ELectrostatic Potentials on a Grid),^11^ Merz‐Kollman,^12^ Stout‐Politzer,^65^ Restrained Electrostatic Potential (RESP),^51^ Bickelhaupt,^36^ Bader Quantum Theory of Atoms in Molecules (QTAIM)^1^
Gaussian 16^59^	Mulliken,^14^ Hirshfeld,^20^, ^21^ Iterative Hirshfeld,^22^ HLY (Hu−Lu‐Yang),^13^ Minimal Basis Set Mulliken,^37^ Natural Population Analysis (NPA),^15^ Charge Model 5 (CM5),^29^ Atomic Polar Tensor Charges (APT)^52^
MOLPRO 2019^60^	Intrinsic Bond Orbitals (IBO)^16^
Horton 3^66^	Iterative Stockholder Analysis (ISA),^23^ Minimal Basis Set Iterative Stockholder Analysis (MBIS)^25^
ACP^67^ and I‐ACP^67^	Adjusted Charge Partitioning (ACP),^54^ Iterative Adjusted Charge Partitioning (I‐ACP)^55^
DFTD4^43^	Electronegativity Equilibration Charges (EEQ)^68^
DDEC6^24^	DDEC6 (density derived electrostatic and chemical approach, version 6)^24^
IBOView^16^	Fuzzy Voronoi^46^ (graphical user interface only)

Principal component analysis was initially performed upon 25 different population analysis methods. Our initial analysis identified some near‐redundancies (see below), leading to some winnowing.

Upon completing the analysis, its statistical stability was tested by adding elements that were randomly chosen from the dataset to provide noise. The structure of principal components along with the explained variance values was compared between the untouched and adulterated data to assess how robust the implied structure markers within the data are.

## 2 Results and Discussion

The full correlation matrix of all variables, as well as the full partial charges dataset, are given in Microsoft Excel format in the Supporting Information.

One thing becomes immediately clear if the matrix is blocked by charge distribution type: blocks with very high correlation coefficients within them emerge. Keeping all of these around in the principal component analysis leads to a large number of near‐zero eigenvalues.

By way of illustration, we show here the block between the four electrostatic charge variants:


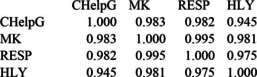


Its eigenvalues and eigenvectors (i. e. the principal components of the correlation matrix) are:


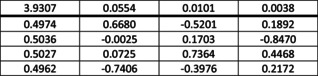


Note that the eigenvalues of a correlation matrix add up to its dimension, *in casu*, four. The largest eigenvector (effectively the arithmetic mean of all four variables) has eigenvalue 3.93, the next largest (corresponding to CHELPG vs. HLY) having eigenvalue 0.055. One naive variable selection technique, discussed by Jolliffe^69^ in Section 9.3 of his textbook on Principal Component Analysis, is to eliminate the variables that have the largest coefficient in the eigenvectors with the lowest eigenvalues. This ‘backward elimination’ (BE) strategy leads to Merz‐Kollman and RESP being eliminated, leaving just CHELPG and HLY. The eigenvectors for the remaining 2×2 correlation matrix are just (CHELPG±HLY)/√2. Whether one chooses to retain CHELPG or HLY for further analysis is basically arbitrary: CHELPG is available in a larger number of codes, while HLY is implemented (in Gaussian) for all elements in the Periodic Table. In the present paper, we have elected to retain HLY.

Between the four iterative Class IIb1 methods, we have the following correlation matrix:


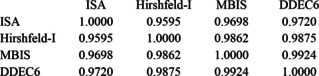


Its largest two eigenvalues are 3.934 and 0.045, the former corresponding to all charges moving in tandem and the latter to ISA pulling in the opposite direction from the three others. Within the lower 3‐variable block, the top eigenvalue is 2.977, showing that these three charge types on the whole contain very similar information.

The squared correlation matrix for 18 variables has been given in Table 2, reordered to maximize blocking. Some comments concerning it are in order here:

**Table 2:** The squared correlation matrix for a subset of all variables. For clarity, the variables have been ordered to maximize blocking. The color gradient runs from white for R^2^≤0.80 to deep blue for R^2^≥0.98.

	VDD	Hirshfeld	HLY	ISA	DDEC6	MBIS	Hirshfeld‐I	MBS‐ Bickelhaupt	NPA	IBO	MBS‐ Mulliken	i‐ACP	ACP	CM5	EEQ	QTAIM	APT	ADCH
VDD	1.00	0.96	0.64	0.78	0.81	0.77	0.78	0.78	0.74	0.78	0.70	0.78	0.78	0.76	0.73	0.64	0.68	0.68
Hirshfeld	0.96	1.00	0.67	0.79	0.85	0.80	0.80	0.79	0.78	0.82	0.76	0.75	0.80	0.78	0.73	0.61	0.62	0.72
HLY	0.64	0.67	1.00	0.87	0.84	0.84	0.79	0.76	0.73	0.73	0.72	0.71	0.77	0.73	0.70	0.48	0.51	0.68
ISA	0.78	0.79	0.87	1.00	0.94	0.94	0.92	0.89	0.81	0.80	0.77	0.89	0.85	0.78	0.77	0.69	0.74	0.69
DDEC6	0.81	0.85	0.84	0.94	1.00	.99	0.98	0.94	0.92	0.91	0.90	0.85	0.89	0.84	0.81	0.66	0.67	0.75
MBIS	0.77	0.80	0.84	0.94	0.99	1.00	0.97	0.95	0.93	0.89	0.90	0.86	0.90	0.84	0.82	0.66	0.66	0.74
Hirshfeld‐I	0.78	0.80	0.79	0.92	0.98	0.97	1.00	0.94	0.92	0.89	0.88	0.87	0.86	0.80	0.78	0.74	0.73	0.68
MBSBickelhaupt	0.78	0.79	0.76	0.89	0.94	0.95	0.94	1.00	0.92	0.87	0.90	0.89	0.92	0.87	0.86	0.75	0.71	0.71
NPA	0.74	0.78	0.73	0.81	0.92	0.93	0.92	0.92	1.00	0.96	0.98	0.73	0.85	0.84	0.81	0.60	0.55	0.73
IBO	0.78	0.82	0.73	0.80	0.91	0.89	0.89	0.87	0.96	1.00	0.96	0.71	0.81	0.82	0.80	0.56	0.55	0.75
MBSMulliken	0.70	0.76	0.72	0.77	0.90	0.90	0.88	0.90	0.98	0.96	1.00	0.68	0.84	0.85	0.83	0.52	0.49	0.76
i‐ACP	0.78	0.75	0.71	0.89	0.85	0.86	0.87	0.89	0.73	0.71	0.68	1.00	0.88	0.77	0.77	0.84	0.83	0.59
ACP	0.78	0.80	0.77	0.85	0.89	0.90	0.86	0.92	0.85	0.81	0.84	0.88	1.00	0.91	0.89	0.63	0.60	0.75
CM5	0.76	0.78	0.73	0.78	0.84	0.84	0.80	0.87	0.84	0.82	0.85	0.77	0.91	1.00	0.97	0.51	0.48	0.81
EEQ	0.73	0.73	0.70	0.77	0.81	0.82	0.78	0.86	0.81	0.80	0.83	0.77	0.89	0.97	1.00	0.54	0.50	0.76
QTAIM	0.64	0.61	0.48	0.69	0.66	0.66	0.74	0.75	0.60	0.56	0.52	0.84	0.63	0.51	0.54	1.00	0.86	0.36
APT	0.68	0.62	0.51	0.74	0.67	0.66	0.73	0.71	0.55	0.55	0.49	0.83	0.60	0.48	0.50	0.86	1.00	0.36
ADCH	0.68	0.72	0.68	0.69	0.75	0.74	0.68	0.71	0.73	0.75	0.76	0.59	0.75	0.81	0.76	0.36	0.36	1.00

- Note the high correlation (R^2^=0.96) between VDD and ordinary/original Hirshfeld. Both are based on partitioning the same promolecular density: the key difference is of course that Hirshfeld uses fuzzy boundaries derived from ρ_proatom_/ρ_promolecule_, while VDD employs discrete Voronoi tesselation. Apparently, the impact of this difference on the statistical similarity of the variables is smaller than one might naively expect.
- In contrast, squared correlations between ordinary Hirshfeld and the various iterative Class IIb1 methods are surprisingly small, around 0.80 except for DDEC6 (R^2^=0.85). Indeed, ISA has a greater correlation with HLY electrostatic charges (R^2^=0.87), as does MBIS (R^2^=0.84); DDEC6’s R^2^ with HLY is similar to its correlation with ordinary Hirshfeld.
- The iterative Class IIb1 methods emerge as a block, out of which ISA “only” has R^2^=0.92–0.94 with the others, but the remaining three have no R^2^ below 0.97 between them. ACP and i‐ACP can be seen as peripheral members of the block: in light of the conceptual similarity between MBIS and i‐ACP, the relatively low R^2^[MBIS, i‐ACP]= 0.86 is somewhat surprising. Rather more surprisingly, this latter sub‐block of DDEC6, MBIS, and Hirshfeld‐I has R^2^=0.94–0.95 with MBSBickelhaupt, that is, Bickelhaupt's modified Mulliken after minimal basis set projection. (While R^2^[DDEC6,MBIS]=0.99 and R^2^[DDEC6,Hirshfeld‐I]=0.98 suggest great similarity, these coefficients of determination would likely have been lower for a more solid state/materials science oriented sample, where DDEC6 should be more resilient.)
- MBSMulliken, NPA, and IBO form a block with all R^2^≥0.96, and indeed R^2^ between NPA and MBSMulliken reaches 0.98. Of these three, NPA has the largest cross‐correlations with iterative Class IIb1 methods (other than ISA), and IBO the smallest. MBSBickelhaupt correlates slightly less well with NPA and IBO, but better with the iterative Class IIb1 methods.
- CM5 and EEQ exhibit a surprisingly large R^2^=0.97 between them; this might be ascribed to the design of CM5 as an empirical correction to Hirshfeld for better electrostatics. However, the correlation between either method and HLY, for instance, is fairly low (R^2^=0.70 for EEQ, 0.73 for CM5) while rather better correlations are found with some of the iterative Hirshfeld variants, and particularly with ACP. Somewhat surprisingly, since ADCH like CM5 was designed as a correction to Hirshfeld for better properties, ADCH correlates less well with CM5 than one might expect (R^2^=0.81).
- QTAIM has relatively high R^2^={0.86,0.84} with {APT,i‐ACP}, but much smaller ones with the “Hirshfeld” and “NPA” blocks, or with the electrostatic blocks.
- i‐ACP acts as a kind of ‘bridge’ to the various iterative Hirshfeld flavors, with R^2^=0.85‐0.89. The older ACP, on the other hand, correlates less well with the Hirshfeld family but rather better, R^2^=0.89 and 0.91, with EEQ and CM5, respectively.

Principal component analysis (PCA) on the correlation matrix reveals that the first two principal components, with eigenvalues 15.93 and 0.82, respectively, account for most of the variation in the dataset. Let us now switch to PCA on the covariance matrix.(Table 3)

**Table 3:** First six principal components found after PCA on the covariance matrix.

Eigenvalues	2.145	0.157	0.072	0.022	0.017	0.010
VDD	−0.08	0.00	−0.01	0.15	0.22	−0.26
Hirshfeld	−0.08	0.02	−0.02	0.12	0.18	−0.27
CHELPG	−0.21	0.01	0.37	0.04	−0.03	−0.03
MK	−0.21	0.11	0.39	−0.08	−0.11	−0.08
RESP	−0.21	0.10	0.37	−0.05	−0.09	−0.11
HLY	−0.22	0.20	0.38	−0.16	−0.28	−0.14
ISA	−0.23	0.03	0.21	−0.07	0.14	0.12
DDEC6	−0.21	0.09	−0.01	−0.09	0.10	0.10
MBIS	−0.26	0.11	−0.02	−0.13	0.02	0.32
Hirshfeld‐I	−0.26	0.03	−0.08	−0.23	0.06	0.22
MBSBickelhaupt	−0.26	0.02	‐0.14	0.12	−0.08	0.25
NPA	−0.28	0.20	−0.35	−0.22	−0.05	0.01
IBO	−0.21	0.17	−0.23	−0.16	0.16	−0.35
MBSMulliken	−0.28	0.29	−0.37	−0.15	−0.07	−0.08
i‐ACP	−0.20	−0.13	0.07	0.28	0.09	0.32
ACP	−0.14	0.06	−0.02	0.31	0.01	0.21
CM5	−0.14	0.13	−0.05	0.43	0.01	0.10
EEQ	−0.14	0.11	−0.06	0.46	−0.02	0.09
QTAIM	−0.38	−0.72	−0.18	0.05	−0.47	−0.24
APT	−0.24	−0.40	0.09	−0.14	0.71	−0.02
ADCH	−0.13	0.20	−0.02	0.37	0.12	−0.49

Normalizing the eigenvalues by the number of variables, we find that the first three eigenvectors (i. e., the first three principal components) contain as much variation as 20 out of the total 21 primitive variables.

The first eigenvector, PC_1_, has all charges pulling in the same direction with different strengths: PC_1_ can thus be said to correspond to Schwarz and Meister's^7^ ‘principal component of ionicity’. The different coefficients correspond roughly to how pronounced charge differences are: e. g., for the original Hirshfeld we see 0.08, for iterative Hirshfeld 0.26, for DDEC6 0.21, and for QTAIM 0.38.

PC_2_ is another matter: it pits QTAIM, APT, and i‐ACP with same signs against every other distribution with opposite sign. PC_2_ can hence be seen as a secondary “principal component of ionicity”. Why APT, despite its different physical principles, marches in comparative lockstep with QTAIM is somewhat opaque to the authors.

PC_3_, which may still be of marginal statistical significance, pits the electrostatic charges and ISA against the orbital‐based charges.

In order to verify how well determined PC_2_ and PC_3_ are, about 10 % random numbers were added to the sample and the analysis repeated. The eigenvalue for PC2 clearly stays in well‐defined territory, but PC_3_ appears to be in danger of ‘drowning in the noise’. We therefore caution against overinterpreting PC_3_.

What happens if we retain just one representative of each “physical” group? This leads to the following:


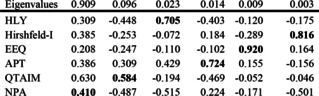


Principal component analysis is one way to address the feature selection problem. PCs still require calculating or measuring all the variables, however: hence, in data science and machine learning, some attention has been devoted to the variable selection problem, i. e.: to extracting the subset of original variables that contains most of the information in the dataset. A simplistic way is ‘hunting’ for the variables with the largest coefficients in the PCs, but Jolliffe's textbook on PCA^69^ cautions against this. Ref. [70] discusses more sophisticated approaches and their implementation in the subselect module of the R statistical software system.^71^

“Subselect” works through simulated annealing to optimize a choice of objective functions.^70^ We ended up using the GCD (generalized coefficient of determination) criterion, that is, the overlap between the subspace spanned by variable subset size n and that spanned by first n principal components. (The analysis was run using R version 3.6.1 on the senior author's Macbook Pro.)

However, if we apply *subselect* to the covariance matrix of the various population types, we find that the objective function goes through a maximum for 3 variables, and that these are MBSMulliken, QTAIM, and HLY. (Solutions using another electrostatic potential charge instead of HLY, or NPA instead of MBSMulliken, are essentially of the same quality.) For a single variable, we get Hirshfeld‐I as the optimum; for two variables, MBSMulliken and QTAIM. Expanding to four and five variables successively adds EEQ and APT, respectively.

If one forces inclusion of Hirshfeld‐I, then successive additions lead to (again, using the GCD criterion):

2 variables: Hirshfeld‐I+QTAIM

3 variables: Hirshfeld‐I+QTAIM+ISA

4 variables: Hirshfeld‐I+QTAIM+ISA+EEQ

Beyond which the “mold is broken”:

5 variables: Hirshfeld‐I+QTAIM+EEQ+HLY+APT

However, multiple regression in terms of (MBSMulliken, HLY, QTAIM) reveals that Hirshfeld‐I can be reproduced at R^2^=0.961 by 0.252 HLY+0.432 MBSMulliken+0.205 QTAIM. In other words, if those other three variables are present then Hirshfeld‐I is effectively redundant.

Intriguingly, said three variables correspond to one representative each of the three Corminboeuf classes.

Let us consider the eigenvalues and eigenvectors of the covariance matrix for this 3‐variable subset:


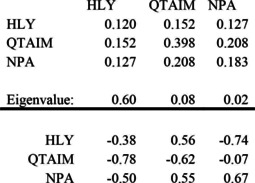


The structure here is very clear: (1) Principal ionicity; (2) QTAIM in opposition to the others; (3) Electrostatic versus orbital.

Another angle on the issue is afforded by considering the squared Pearson correlations R^2^ for least‐squares fits of the individual charges in terms of the first few principal components. The result is shown in Table 4.

**Table 4:** R^2^ for least‐squares fits of the individual charges as linear combinations of the first few principal components.

	C(PC_1_)	C(PC_2_)	C(PC_3_)	R^2^(PC_1_)	R^2^(PC_1, 2_)	R^2^(PC_1,2,3_)
Hirshfeld	0.085	0.027	0.017	0.8092	0.8158	0.8164^[e]^
CM5	0.151	0.145	0.019	0.8348	0.8981	0.8983^[c]^
Hirshfeld‐I	0.278	0.052	0.007	**0.9752**	0.9780	0.9780
NPA	0.304	0.253	−0.342	0.9160	0.9680	*0.9921*
HLY	0.229	0.193	0.601	0.7959	0.8419	0.9552^[a]^
ISA	0.252	0.032	0.404	0.9362	0.9338	0.9835
MBIS	0.277	0.137	0.111	0.9629	*0.9821*	0.9853
DDEC6	0.227	0.108	0.104	0.9638	*0.9818*	0.9860
MBSMulliken	0.301	0.340	−0.365	0.8766	0.9677	**0.9943**
EEQ	0.155	0.122	−0.003	0.8297	0.8718	0.8718^[d]^
QTAIM	0.419	−0.699	−0.274	0.8022	**0.9842**	*0.9913*
IBO	0.226	0.201	−0.216	0.8913	0.9492	0.9661
ACP	0.155	0.075	0.069	0.9007	0.9180	0.9216
i‐ACP	0.213	−0.121	0.185	0.9207	0.9450	0.9594
APT	0.258	−0.401	0.187	0.7774	0.9303	0.9387^[b]^
MBSBickelhaupt	0.281	0.047	−0.075	**0.9765**	0.9787	0.9801

[a] R^2^=0.9972 with 6 components: C(PC4)=−0.080, C(PC5)=−0.470,C(PC6)=+0.470. [b] R^2^=0.9938 with 5 components: C(PC4)=−0.328, C(PC5)=0.666. [c] R^2^=0.9801 with 4 components: C(PC4)=0.455. [d] R^2^=0.9681 with 4 components: C(PC4)=0.509. [e] R^2^=0.9324 with 7 components, R^2^=0.9927 with 10 components.

We note that the first principal component has the strongest correlation with Hirshfeld‐I (R^2^=0.9752) and with MBSBickelhaupt (R^2^=0.9765), while MBIS and DDEC6 (both iterative Hirshfeld variants) come quite close at R^2^=0.963 and 0.964, respectively. The effect of adding the second principal component is essentially negligible for Hirshfeld‐I and MBSBickelhaupt (both of which have small loadings in PC2) but QTAIM is now the winner at R^2^=0.9842, albeit closely followed by the iterative Class IIb1 methods other than ISA. If we consider the largest increases in R^2^ from one to two PCs, then QTAIM followed by APT seems to be most associated with PC2. The corresponding increase from adding PC3 is by far the largest for HLY, which jumps from 0.84 to 0.95. To reach 0.99 territory, HLY requires adding in PC5 and PC6: its loading in PC4 is too small to be useful. CM5 and EEQ would benefit from a 4^th^ PC.

From the converse point of view, we may consider how well the three first PCs would be expressed as linear combinations of the three variables (MBSMulliken or NPA, QTAIM, HLY). The R^2^ for these fits are 0.990 for PC1, 0.977 for PC2, and 0.843 for PC3. Increasing the latter number further (as well as good fits for PC4 and PC5) can be achieved by adding EEQ and APT.

## 3 Conclusions

From a principal component analysis of about two dozen different charge distributions for a sample of over 2,000 main‐group molecules, we can establish the following.

1. There are two very well‐defined “principal components of ionicity” PCq1 and PCq2, and a more weakly defined third component PCq3.
2. The trio of individual charge distributions that best describes the space covered by PCq1, PCq2, and PCq3 is QTAIM with MBSMulliken (or NPA) and HLY (or another electrostatic potential charge) — in other words, one representative each of the three classes of Corminboeuf et al. If we omit PCq3, then HLY (or other electrostatic charge) can be omitted.
3. For the single charge distribution that most closely resembles PCq1, Hirshfeld‐I and MBS‐Bickelhaupt are effectively tied, both with R^2^>0.975. If QTAIM, MBSMulliken, and HLY are included, however, Hirshfeld‐I can be represented as their linear combination with R^2^>0.96: Hirshfeld‐I ≈0.252 HLY+0.432 MBSMulliken+0.205 QTAIM.
4. The “first principal component of ionicity” PCq1 corresponds to the one posited by Schwarz and Meister: all charges move in concert at different amplitudes.
5. The “second principal component of ionicity” PCq2 corresponds to the difference between QTAIM‐type charges and other charge types, except that (intriguingly, in view of the different physics) APT somewhat resembles QTAIM.
6. The more weakly defined PCq3 corresponds to the opposition between electrostatic and orbital‐based charges, leaving QTAIM nearly unaffected. Further components are statistically too weakly defined.
7. Most types of partial charges can be fitted quite well as linear combinations of PCq1, PCq2, and PCq3, though some require a fourth of fifth principal component for a good fit. Intriguingly, original Hirshfeld is the most resistant to expansion in principal components, requiring as many as ten of them.
8. Behavior of IBO (intrinsic bond orbital) charges is statistically very similar to NPA (natural population analysis) as well as MBSMulliken, that is, Mulliken population analysis after minimal basis set projection.

Somewhat surprisingly, discrete vs. fuzzy partitioning of the deformation density (i. e., Voronoi Deformation Densities vs. original Hirshfeld charges) causes a much smaller difference than iterating the pro‐atomic charges that make up the promolecular density.

We also define a minimal basis set‐projected variant of Bickelhaupt charges, which should be easy to implement in any electronic structure system, or postprocessing code for same, for which one has source code available.

In all, no single charge distribution tells the whole story, but a trio of well‐defined ones – i.e., one representative each of the three major classes of Corminboeuf and coworkers – should be able to cover most aspects of it.

## Conflict of interest

The authors declare no conflict of interest.

## Supporting information

As a service to our authors and readers, this journal provides supporting information supplied by the authors. Such materials are peer reviewed and may be re‐organized for online delivery, but are not copy‐edited or typeset. Technical support issues arising from supporting information (other than missing files) should be addressed to the authors.

Supplementary

## Footnotes

1 F. L. Hirshfeld himself used the term “stockholder population analysis” for what is today commonly known by his last name.

2 Among many other things, proatom densities are evaluated in the presence of compensating background charges.
